# Training load and health problems in freshman rowers

**DOI:** 10.1186/2052-1847-7-S1-O1

**Published:** 2015-08-11

**Authors:** Anne-Marie van Beijsterveldt, Mark de Jong, Koen Lemmink, Janine Stubbe

**Affiliations:** 1 Netherlands Organization for Applied Scientific Research (TNO), Leiden, the Netherlands; 2 Centre for Human Movement Sciences, University Medical Centre Groningen, University of Groningen, Groningen, the Netherlands; 3 School of Sports Studies, Hanze University Groningen, University of Applied Sciences, Groningen, the Netherlands; 4 Amsterdam University of Applied Sciences, School of Sports and Nutrition, Amsterdam, the Netherlands; 5 Codarts University for the Arts, Rotterdam, the Netherlands

## Background

Rowing is a popular sport for students in the Netherlands. First-year students have to deal with a substantial increase of training exposure during their rowing season. The aim of this study was to investigate the training characteristics and the occurrence of injuries and illnesses in the freshman rowers.

## Methods

Novice rowers of 5 Dutch student rowing clubs were prospectively followed during the season 2013-2014. Prior to the start of the season, all participants filled in a baseline questionnaire about anthropometric characteristics, injury history and rowing experience. During the 7 months follow up, an online questionnaire was filled in on a weekly basis to monitor exposure (duration and intensity of training sessions and races) and health (injuries and illnesses). To collect this information the OSTRC Overuse Injury Questionnaire was used (Clarsen et al., 2013).

## Results

In total, 137 freshman rowers took part in this study (63% man, 37% women; mean age 20.4± 1,5 years). Preliminary results show that 3122 questionnaires were filled in during the season (mean = 23, median = 26, range 1-34 per rower). On average, the rowers spent more than 7 hours (430 minutes) of training per week and they performed on average 2.9 race kilometers per week. The mean intensity of rowing was assessed as “somewhat hard – hard”, 14 on a scale of 6 – 20 (= Rate of Perceived Exertion).

In almost 4 out of 10 questionnaires (37%) problems during rowing in the past week were registered. In 28% of all questionnaires symptoms or health complaints during the past week were mentioned. Injuries and illnesses were the most prevalent types of these health problems (56% and 31% respectively). Eighty percent of the rowers (n=109) sustained 1 (or more) injuries during the season. The most common injury locations were knee (30%) and lower back (17%).

## Conclusions

The injury/illness incidence is high for freshman rowers. Nevertheless, the current knowledge on the epidemiology of rowing injuries and illnesses in novice rowers is scarce. Our results can form the starting point for further research on risk factors and injury mechanisms. Finally, effective injury and illness prevention programs for rowers are needed.
